# Agro-pastoralists’ perception of climate change and adaptation in the Qilian Mountains of northwest China

**DOI:** 10.1038/s41598-022-17040-2

**Published:** 2022-07-25

**Authors:** Shuntao Xie, Wenguang Ding, Weifeng Ye, Zhe Deng

**Affiliations:** 1 College of Earth and Environmental Sciences, Lanzhou University, Lanzhou, 730000 China; 2 Present Address: Key Laboratory of Western China’s Environmental Systems (MOE), Lanzhou University, Lanzhou, 730000 China

**Keywords:** Climate change, Climate sciences, Ecology, Environmental sciences, Environmental impact, Climate-change adaptation, Climate-change impacts, Environmental impact

## Abstract

Global climate change affects all aspects of human society, especially agricultural and animal husbandry production. Northwest China has been detrimentally affected by the climatic variations due to its high exposure to extreme climatic events. A number of studies have reported agro-pastoralists’ perceptions and adaptation responses to climate change, but the current knowledge of agro-pastoralists’ perceptions of climate change in China are insufficient. To fill this research gap, this study aims to investigate the perception level of agro-pastoralists in Northwest China on climate change and related factors. Data were collected using a structured questionnaire based on household surveys of 554 study participants in four counties in Gansu Province, China. Raw data were collected using stratified random sampling. A probit model was used to analyze the respondents' understanding of climate change and its related socio-economic and demographic variables. Our results show that the majority of respondents were aware (70%) of the changes in temperature and precipitation. Socioeconomic and demographic variables such as gender, farming experience, education level, cultivated land size, agricultural income, livestock, village cadre experience, access to weather information of agro-pastoralists are pertinently related to agro-pastoralists’ awareness of climate change. Farming experience, education level, household size, grassland size, agricultural income, association membership, village cadre experience has a high impact on agro-pastoralists' adaptation to climate change. The results of this study will help guide government agencies and decision makers, and help arid and semi-arid areas to build sustainable adaptation measures under the framework of climate change. The study recommends institutions targeting households’ livelihood improvement and making decisions concerning climate change adaptation need to focus on mass media and information technology, improving locally adapted extension services, improved irrigation, expand loan channels.

## Introduction

Climate change includes many factors that affect human systems in different ways^1^. Growing evidence indicates that climate is changing, prompting increasing efforts to understand the possible impacts of these changes^2,3^. In addition, agriculture is recognized as one of the most vulnerable sectors to climate change^4–7^. Therefore, the impact of climate change on agriculture must be taken seriously.

The IPCC Special Report on Climate Change and Land noted that agriculture and food system will be increasingly affected by expected future climate change^8^. The most important and direct impact of climate change on agriculture is the negative impact on crop yield. Under the most conservative climate change scenario, net grain production in Asia is expected to decline by 3% to 12% by the end of the twenty-first century^9^. For China, under scenarios B2 and A2 of the special report on emission scenarios^1^, the productivity of major staple crops, maize, wheat and rice may decline by 26.9–36.4%, 12.9–21.7% and 12.4–28.6%, respectively^10^.

The IPCC^1,8^ reported that the continuous intensification of greenhouse effect caused by human activities and greenhouse gas emissions are one of the important causes of global warming. Research shows that China's climate warming is basically synchronous with global warming, but there are significant regional and seasonal differences^1,11–13^. In the face of these changes, it is of practical significance to study the characteristics and trends of temperature change in a certain region in helping, to understand the characteristics of climate change in the region, to predict meteorological and climatic disasters, to rationally develop and utilize climate resources, and to improve the production environment of agriculture and animal husbandry.

Some of the most catastrophic effects of global climate change on people's livelihoods will be in countries with a large agricultural population that are ill-equipped to cope with the social and political pressures associated with migration and deteriorating livelihoods. Many developing countries face similar challenges, and many case studies in these regions focus on and document the impact of climate change on livelihoods. Because these populations rely heavily on rain-fed agriculture, the effects of increasing weather change are amplified^14–18^.

Studies of perception are important to support risk analysis and adaptive responses. The perception of climate change varies from person to person, and individuals' awareness and belief about climate change and their political orientation will affect the perception of climate change^19–23^. Although the way people perceive climate change and their personal understanding may not be accurate or complete, the perception and understanding of climate change can improve the effectiveness of climate change adaptation^24–27^. Climate change adaptation is obviously regional and non-replicable, and there are local differences in adaptation capacity and adaptation resources^28–31^. Therefore, "bottom-up" adaptation research is more targeted and more practical for local governments to carry out adaptation management and adaptation technical guidance^32–34^. In the "bottom-up" experimental research, scholars have carried out many targeted studies based on the "perception-adaptation" analytical framework^25,35,36^, perception is the premise and basis of adaptation decision and choice, but the final adaptation decision and action is also affected by various social, economic, environmental and individual factors. For example, Deressa et al.^20^ studied farmers' adaptation to climate change in The Nile Basin of Ethiopia and showed that local farmers' adaptation actions were mainly affected by information access, gender, age, education level and other factors. The study of Abid et al.^37^ on Punjab Province in Pakistan showed that education, farming experience, family size, land area, access to market information and the ability to obtain weather forecast information were the main factors affecting the selection of adaptation measures by local farmers. Addisu et al.^38^ found that age, education level, assets, extended agricultural services and distance from health centers were significant influencing factors for local climate change adaptation decisions through investigation and analysis of rural residents in Tana Basin lake region of Ethiopia.

The study area is located in the Qilian Mountains in northwest China, which is a typical area of fragile ecological environment, including the Qilian Mountain National Nature Reserve, also known as the Qilian Mountain National Park. After the establishment of the reserve, the government strictly controlled the number of livestock and strengthened the balance management of grass and livestock, which forced agro-pastoralists to make adaptive changes and reduce the impact of such changes on themselves. Based on the data of Participatory Rural Appraisal (PRA), this paper takes four counties involved in the protected area as the research area, and uses qualitative and quantitative methods. The main research objectives are as follows: (1) assess the perception of climate change by agro-pastoralists in the research area, and whether the perception of climate change is accurate? (2) Analyze the factors affecting agro-pastoralists' perception of climate change; (3) understand the perception of the impact of climate change on agro-pastoralists' livelihood; (4) explore the impact of agro-pastoralists' characteristics on adaptation to climate change.

## Methodology

### Study area information

**Figure 1:**
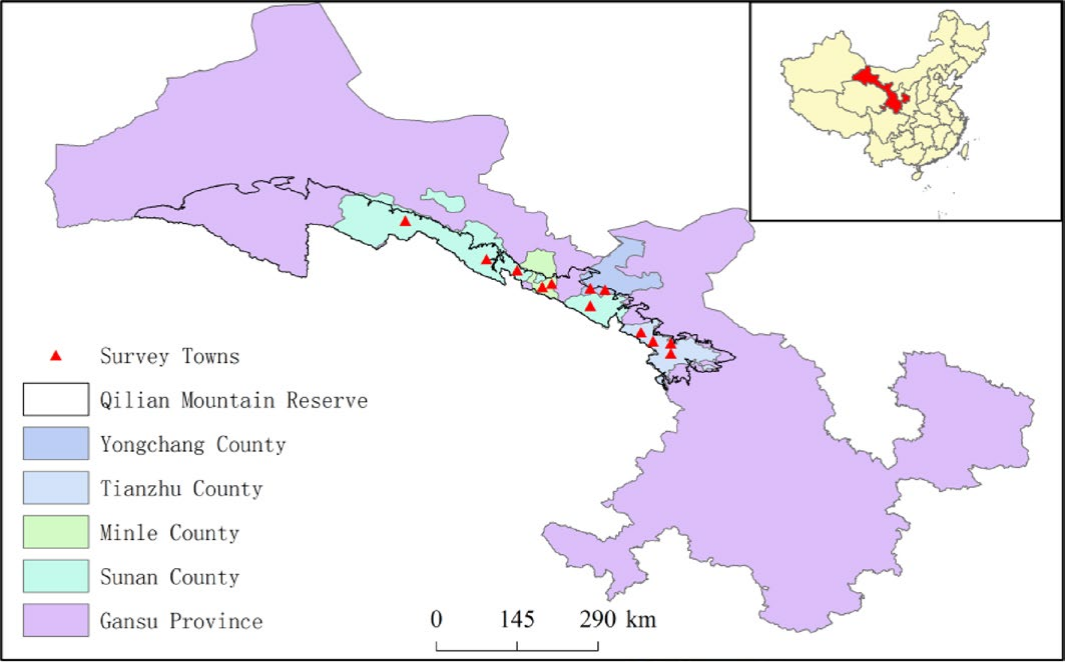
Map of Gansu Province, China showing the survey area. Map of these distinct was drawn by ArcGIS 10.2(https://www.esri.com/).

The research area is located at the northern foot of Qilian Mountain and in the central and western part of Gansu Province. The research focuses on 4 deliberately selected counties and some villages under their jurisdiction, mainly villages near qilian Mountain Nature Reserve (Fig. 1). The altitude of the study area is generally 2000–3500 m above sea level, and Shiyang River, Heihe River, Shule River three major water system are distributed in the area. It is an arid/semi-arid/sub-humid climate with an average annual temperature of 3.6 and an average annual precipitation of 256.1–411.3 mm, which is concentrated in June to September. There are more than 20 ethnic groups, including Han, Yugur, Tibetan, Hui, Mongolian, Tu, Manchu, Dongxiang, Uygur, Hasak and so on.

The main economic activities include: grain crops (wheat, corn, barley, highland barley, beans, potato), cash crops (rape, flaps, beets, Chinese medicinal materials, vegetables, watermelon); livestock (sheep, goats, cattle, pigs, chickens, horses and other domestic animals). Currently, the decrease of grassland growth leads to the decrease of livestock capacity, which indirectly leads to the decline of herdsmen's income level. In addition, the occurrence of some extreme climate events has a dual impact on forage supply and livestock, and may lead to the increase of "white disaster" and "black disaster" and dust disaster. From 1950 to 2009, the study area experienced drought, hail, flood, frost, sandstorm, landslide, debris flow and other extreme weather events, among which drought (55%), hail (17%), frost (6%), sand storm (10%) caused the most serious losses to livestock and crops^39^. The unsynchronization of precipitation and crop growth has brought great challenges to agricultural production. This has prompted government agencies to intervene in the region to raise agro-pastoralists' awareness and adopt climate change risk mitigation strategies through training and capacity-building initiatives. Examples of activities include: adjusting the distribution of crop varieties and planting systems, cultivating drought-tolerant crop varieties, popularizing quantitative grazing, optimizing the structure of herds, strengthening the construction of forage storage warehouses and insulation sheds and other facilities.

### Sample selection

Cross-sectional household survey was conducted by Multistage Random Sampling Techniques. Firstly, three cities involved in the protected area were selected (Administrative divisions at the prefecture level, namely Zhangye, Wuwei, Jinchang); then, one or two county (a city or prefecture comprises several unions) was selected from each city; after that, two or three towns (a county comprises several towns) was selected from each county, and 3 villages were chosen randomly from each towns. Finally, the study covers 3 prefectures, 4 counties, 12 towns, and 34 villages). During the survey, the study followed the sampling strategy of the Household Income and Expenditure Survey (HIES) and interviewed fifteen to twenty farm households from each village as a sample^40^.

The formal investigation took the form of household face-to-face interviews, focusing on interviews with principals and village cadres of specialized agricultural and animal husbandry cooperatives. A total of 34 village-level questionnaires were completed, including 565 sample households, of which 554 households had effective questionnaires, with an effective rate of 98.05%.

The initial research permit was approved by the head of research, including the school, the head of the county government office, the head of the agriculture and Rural Affairs Bureau, the head of the village and the head of the reserve administration, paving the way to seek the consent of the respondents and participants. Voluntary participation was also insisted upon and respondents/participants received detailed information about the study objectives to avoid deception. In the case of interview, participants could choose the place and way of interview so as to avoid the presence of many people or persons in charge. Face-to-face interviews and telephone interviews were conducted according to the participants' own reasons.

All the agro-pastoralists conducted face-to-face interviews at their homes or designated places. In the case of village leaders, face-to-face and telephone interviews were used. Face-to-face interviews took place in homes, shops, workplaces and farms. Interviews lasted about 25 to 35 min and were recorded with the consent of a large group of participants. All the survey interviews agro-pastoralists were conducted in localdialects or minority languages, eventhough some officers used both Mandarin and local dialects.

In addition, before the formal survey, we also selected Huangcheng Town of Sunan Yugur Autonomous County and Haxi Town of Tianzhu Tibetan Autonomous County as the survey areas for the preliminary survey (Table 1), so as to ensure the scientific and practical design of the questionnaire. In addition, in the process of questionnaire survey, centralized inspection and review of each questionnaire should be carried out in time to ensure the completeness and accuracy of questionnaire data.

**Table 1:** Weather stations selected for the purpose of the study.

District	Weather Stations	Altitude (meters)	Available data
Tianzhu	Wushaoling	3045.1	Precipitation (1988–2019) Temperature (1988–2019)
Milne	Milne	2281.4	Precipitation (1988–2019) Temperature (1988–2019)
Yongchang	Yongchang	1976.9	Precipitation (1988–2019) Temperature (1988–2019)
Sunan	Sunan	2311.8	Precipitation (1988–2019) Temperature (1988–2019)
	Jiuquan	1477.2	Precipitation (1988–2019) Temperature (1988–2019)

The structured questionnaire used to collect respondents' information mainly includes five parts: the basic characteristics of agro-pastoralists, awareness of climate change, climate change and disaster effects, and perception and adoption of coping behaviors of agro-pastoralists; village information structured questionnaire mainly includes three parts, including the basic characteristics of the village, climate change and adaptation measures to support and use situation.

### Data analysis

The coding and analysis of qualitative and quantitative data used Microsoft Excel and the Statistical Product and Service Solutions (SPSS version 24.0).The meteorological data include diary records to calculate the average climatic values of seasons, months and years. The normality and outlier test are analyzed by using line diagram, box diagram and scatter diagram. In addition, frequency and percentage were used for descriptive statistics, and demographic independent variables (such as age, farming experience, years of education) and dependent variables (perception) were used for reasoning statistics, such as independent sample t-test and one-way analysis of variance (ANOVA)^14^.Based on the hypothesis, agro-pastoralists' perception of climate change and its impacts, and their socio-economic characteristics are the main factors influencing agro-pastoralists' adaptation choices. Probit model was used to evaluate the impact of selection variables on the respondents' perception and adaptation level. When the independent variables are composed of continuous variables and dummy variables, probit model is particularly suitable for the analysis of binary dependent variables. Assuming that it can predict the impact of independent variables on dependent variables, it is used to evaluate the impact of perception and socio-economic characteristics on climate change adaptation.

### Ethics approval

The Lanzhou University Ethical Committee has approved that this study complies with the ethics of scientific research described in the Ethical Review of Biomedical Research Involving People of China and other applicable ethical principles and legislation in China. Ethical approval was granted by the Lanzhou University Research Administration and Advancement, Research Ethics Sector, Ethical Committee.

### Consent to participate

Respondents volunteered to participate autonomously without their identity being recorded. Informed consent was obtained from all the participants in the study. Consent to participate was voluntary and approved by the Lanzhou University Ethical Committee.

## Results and discussion

### Basic information of interviewees

Results of the descriptive analysis summarized in Table 2 show that more than half of the respondents were males (69%) and were on average 41.3 years old while more than 32 years of farming experience. The study area is comprised of multiple ethnic groups (Han, Tibetan, Yugur, Mongolian, Hui, etc.). In most cases, the main livelihood activity of the Ethnic Minorities (Tibetan, Yugur, Mongolian, Hui, etc.) is livestock, while Han people main livelihood activity is farming. The majority of respondents (64%) were minority nationality. The vast majority of the agro-pastoralists (86%) have a primary school education or above, even though only 1% of them have Undergraduate education or Above. The results also reveal that 92% of respondents have access to weather information. The average cultivated land Per household is 10.23 Mu and Grassland is 156.21 Mu, respectively. The average per household income is RMB78000, and agricultural income is RMB52000.

**Table 2:** Descriptive statistics of agro-pastoralist characteristics.

Variables	Scales	Mean	SD
Gender	1 = Male	0.69	
	2 = Female	0.31	
Age	1 for each year	41.3	15.72
Experience	1 for each year	32.04	14.31
Ethnicity	1 = Han nationality	0.36	
	2 = Minority nationality	0.64	
Education	0 = Illiterate	0.14	
	1 = Primary	0.51	
	2 = Junior	0.23	
	3 = Senior or Adult education	0.11	
	4 = Undergraduate or Above	0.01	
household size	1 for each person	3.87	1.34
Cultivatedland size	Mu	10.23	3.27
Grassland size	Mu	156.1	38.31
Income	Thousand RMB¥	78	21.76
Agricultural income	Thousand RMB¥	52	19.62
Livestock	1 for each livestock	128.3	34.74
Credit loan	0 = No	0.37	
	1 = Yes	0.63	
Insurance	0 = No	0.18	
	1 = Yes	0.82	
Association membership	0 = No	0.42	
	1 = Yes	0.58	
Village cadres	0 = No	0. 87	
	1 = Yes	0.13	
Weather information	0 = No	0.08	
	1 = Yes	0.92	

Due to their long-term farming experience, the agro-pastoralists were expected to have a high-level of understanding of local climate knowledge. Also contributing to this could be the information they receive about climate change and for some, the associated training through agro-pastoralists’ associations. Therefore, they also have a propensity to adapt to adverse conditions resulting from climate change impacts. In addition, the high-level of farming experience, the cultivated-land size, grassland size, Credit loan, Insurance, Village cadres all have a positive impact on the level of agro-pastoralists' adaptation to new climate scenarios.

However, the education level and cadres experience may be the major limiting factors for adopting specific long-term adaptation strategies. Ethnicity and gender are also expected to be key factors influencing awareness and adaptation to climate change. There are differences in relative perception intensity between Ethnic Minority and Han because of their cultural ecology (the main livelihood activity of minorities nationality is livestock, while Han main livelihood activity is farming.). In terms of gender, women in rural areas are less mobile and have less access to information and rights. They are also heavily involved in domestic work. However, men may have easier access to information (socializing, going out to work, etc.) Therefore, male headed households are expected to be more likely to adapt to the impact of climate change.

### Climate change trend in the study area

**Figure 2:**
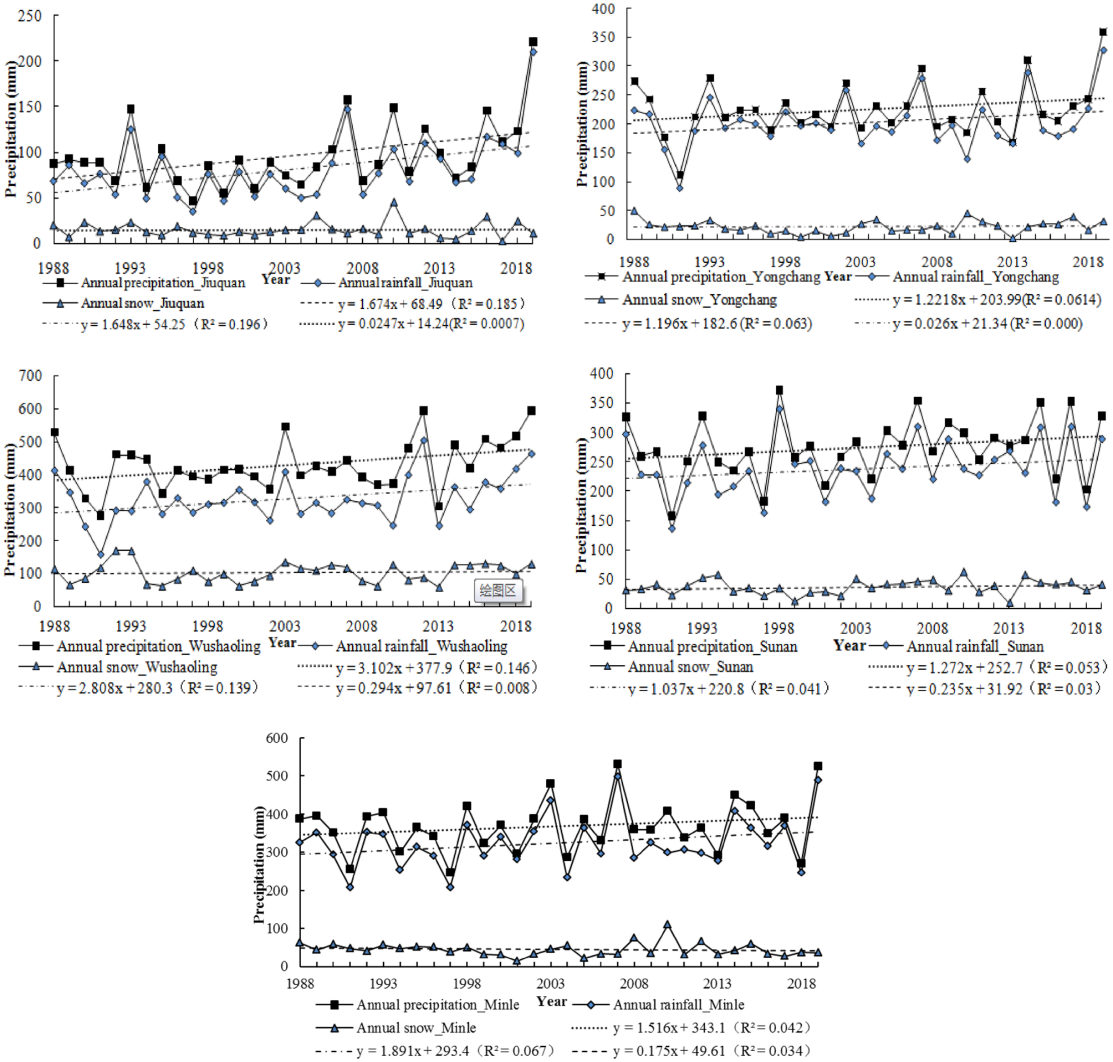
Trends of Annual precipitation, Annual rainfall, Annual snow.

Figure 2 shows the trend of annual precipitation, annual rainfall and annual snow at different meteorological stations in the study area. As shown in the Fig. 2, precipitation, rainfall and snow show an increasing trend, but the increase range of snow (0.0325–0.375/a) is significantly lower than that of precipitation (1.22–3.1/a) and rainfall (1.04–2.81/a). Similarly, through the inspection, it is found that the multi-collinearity among precipitation, rainfall and snow at each meteorological station is obvious (most R^2^ > 0.5, and *p* < 0.01 or 0.05). The oscillation mode of rainfall shows that most of the highest rainfall over the past 32 years occurs in 2019, indicating the wettest year, while 1991 was the driest year over the same period (see Fig. 2). IPCC AR5 pointed out that the global climate system will continue to warm in the future, global precipitation will increase, and water cycles will accelerate, but there are significant regional differences^43^. From 1960 to 2014, the precipitation increased by 6.95 mm/10a^42^, and increased largest in summer^42,43^. Studies have shown that the precipitation and its extreme value increased significantly in the eastern and central part of the Qilian Mountains, and the central was most sensitive to climate warming^42^. IPCC^1,8^ pointed out that climate is the limiting factor affecting agricultural production, especially in inland areas. On the one hand, the temperature fluctuates and rises continuously, the sowing date of crops is advanced, and the growth period of crops becomes longer; On the other hand, the annual fluctuation rate of precipitation increases, and the uncertainty of “rainy year” and “dry year” increases the change of crop yield.

**Figure 3:**
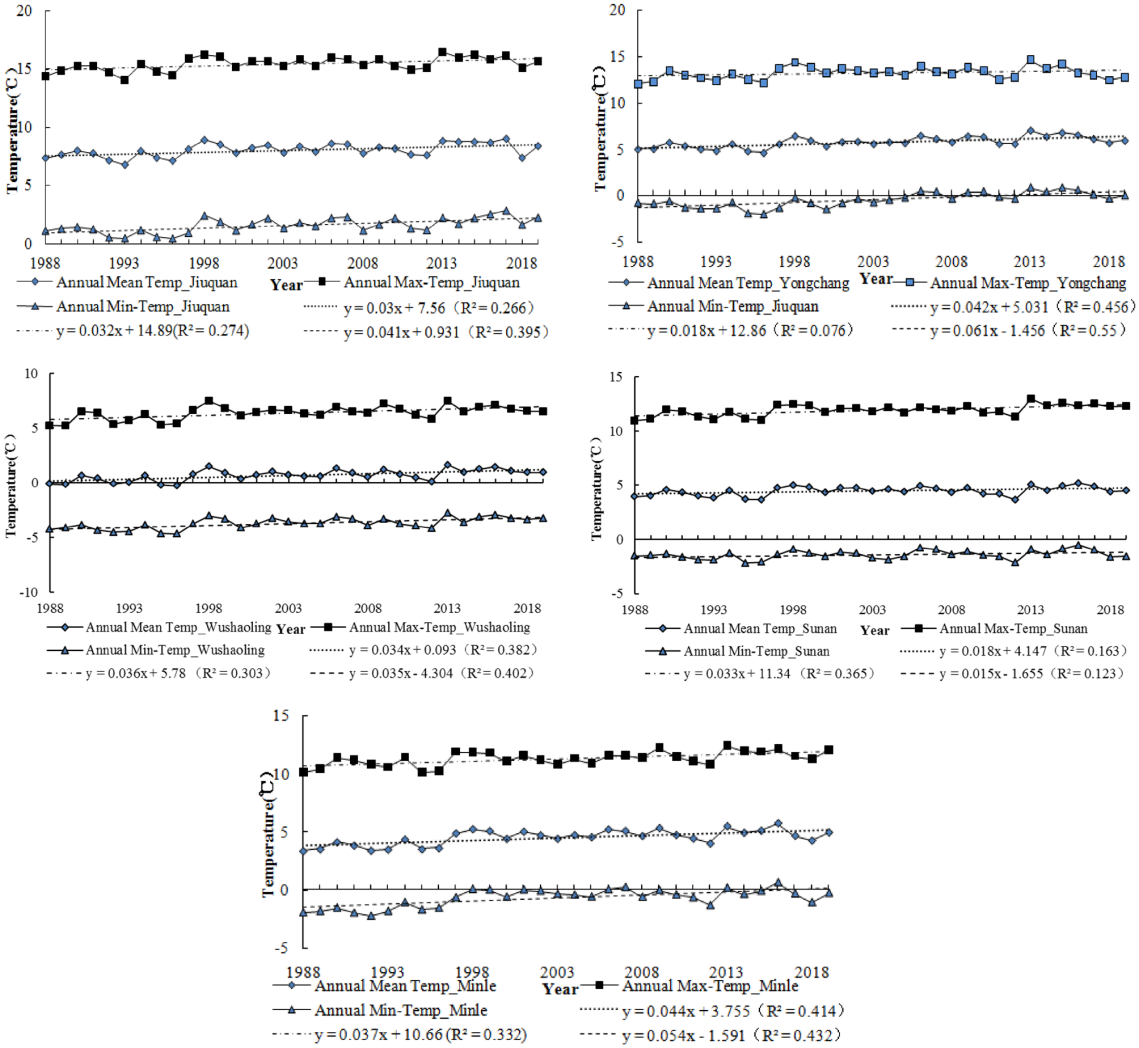
Trends of annual temperature.

In Fig. 3, the trends of annual (mean, max, min) temperature are presented. The results show an increase in mean, maximum and minimum temperature. The annual average temperature increases of each station were 0.3/10a (Jiuquan), 0.42/10a (Milne), 0.34/10a (Wushaoling), 0.18/10a (Sunan) and 0.44/10a (Yongchang), respectively. Overall, this is significant (*p* < 0.01) for the annual mean temperature, annual maximum temperature and annual minimum temperature in the study area.

**Figure 4:**
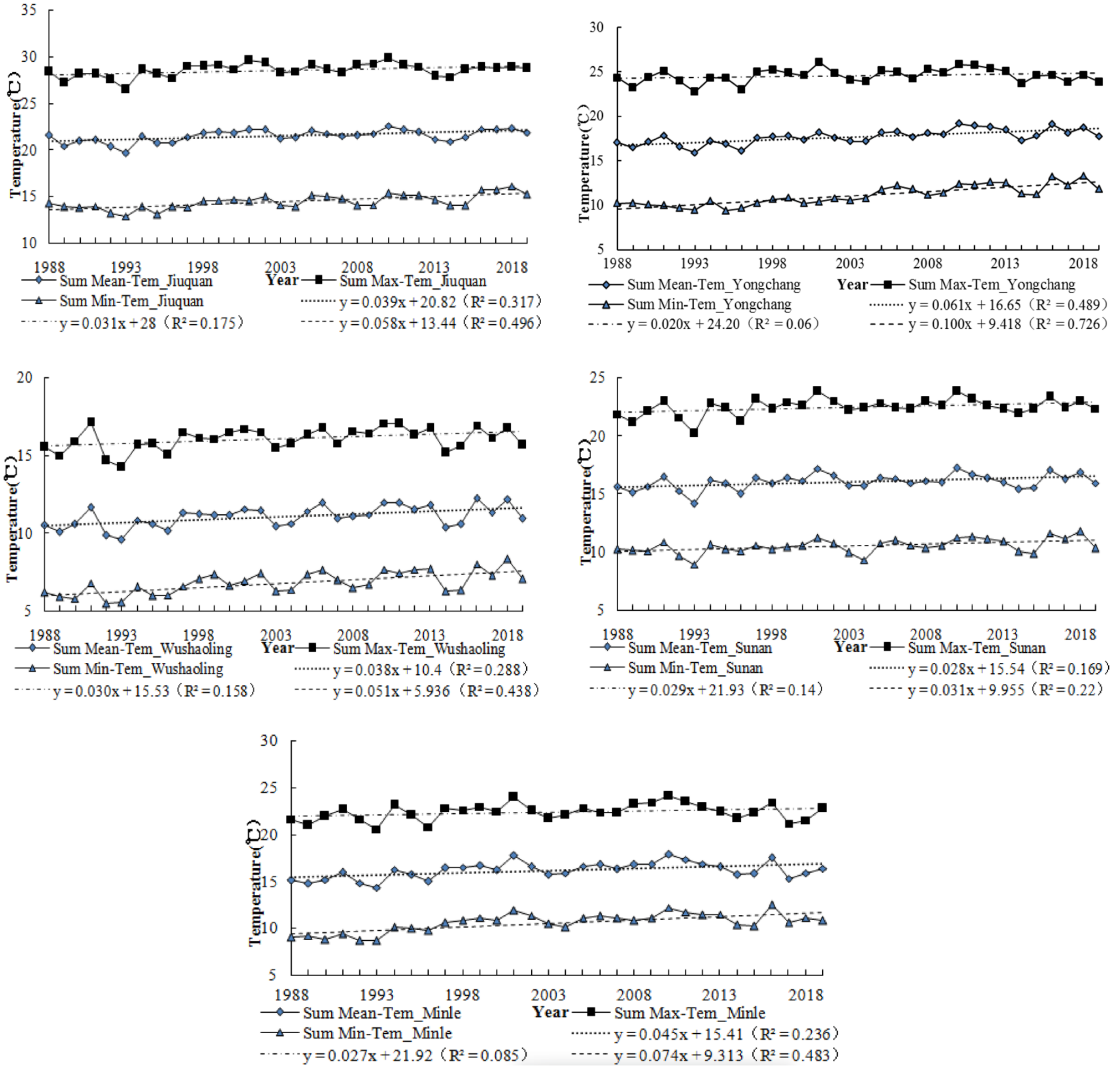
Trends of summer mean temperature.

**Figure 5:**
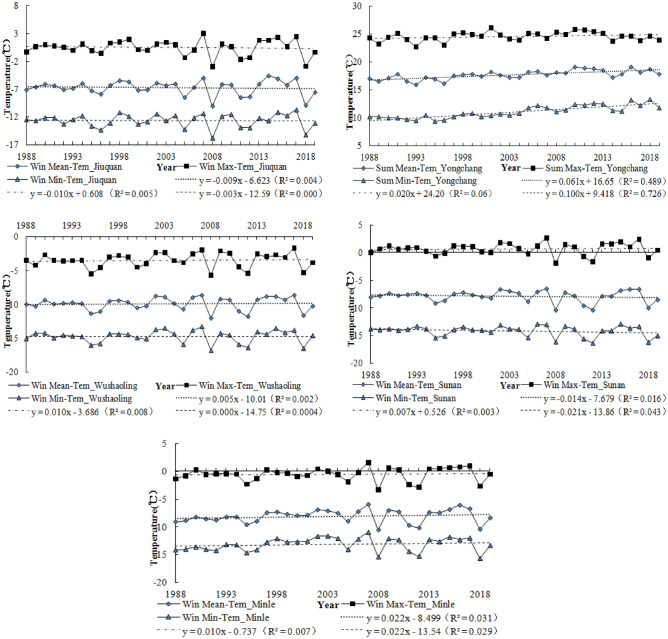
Trends of winter mean temperature.

Figure 4 also shows rising summer (mean, max, min) temperature and Fig. 5 shows rising winter (mean, max, min) temperature. These findings are consistent with previous studies which revealed rising temperature in Qilian Mountains^11–13,45^. The temperature rise provides a favorable environment for carbon 3 and carbon 4 crops, such as wheat, barley, corn and beans. However, the increase of temperature leads to the increase of crop evapotranspiration, which reduces agricultural productivity to a certain extent^8^. The study also reports that rising temperatures have a negative impact on Livestock Reproduction and growth and expose livestock to pasture and water challenges^46,47^. The rampant spread of pests and diseases have also been related to climate change^48–50^. The increasing trend of annual temperature and summer temperature were more significant than winter temperature.

### Respondents’ perception of climate change

The climate change perception of agro-pastoralists is presented in Table 3. Among the 565 agro-pastoralists, 554 had heard about climate change and about 500 believed that climate is changing. Agro-pastoralists understood climate change by different indicators. In the case of temperature, a vast majority of the respondents (83.03%, summer/68.95%, winter) perceived that there have been changes in temperature in the district. Similarly, there have been changes in rainfall in the district as reported by 77.08% (rainfall)/72.74% (snow) of the respondents. In terms of quantity, the majority of the respondents (39.53%) perceive a decrease in rainfall, the next majority of respondents (37.55%) feel that rainfall is increasing. and about 4.69%/7.58% respondents feel that rainfall is unpredictable in terms of quantity (sometimes high, sometimes low).

**Table 3:** Indicators of observed changes in climate.

Variable	Increase	Decrease	Unpredictable	No changes	Don’t know
***A. Precipitation***					
Rainfall	37.55 (208)	39.53 (219)	9.57 (53)	5.78 (32)	7.58 (42)
Snow	47.29 (262)	25.45 (141)	19.13 (106)	3.43 (19)	4.69 (26)
***B. Temperature***					
Summer	71.30 (395)	11.73 (65)	5.23 (29)	8.48 (47)	3.25 (18)
Winter	36.64 (203)	32.31 (179)	14.26 (79)	9.21 (51)	7.58 (42)

Regarding the changes in temperature, the majority of respondents have noticed the rising summer temperature (71.3%), while 11.73% of the respondents perceive that the decreasing summer temperature. For the winter temperature, nearly 32.31% perceive that winter is becoming colder while nearly equal percentage of the respondents (36.64%) perceive that winter is getting warmer. In our study, there are 3.25–7.58% of respondents who do not perceive any changes in temperature; yet this is nearly equal compared to those who did not perceive any changes in rainfall. Climate variables particularly rainfall and temperature have been extensively studied as they are perceived to be significant to the agricultural activities of agro-pastoralists^51–53^.

How well do these perceptions correspond to those estimated by physical measurements? Five sites present similar trends between observed and perceived data: increasing precipitation and temperature (Table 4). In terms of temperature, most respondents (75%, summer /54%, winter) can accurately perceive the temperature rise in the region. Similarly, 63% (rainfall) /66% (snowfall) of the respondents accurately reported the increase rainfall in the region. Gender, farming experience, education level, cultivated land size, agricultural income, livestock, village cadre experience, access to weather information of agro-pastoralists are closely related to agro-pastoralists' awareness of climate change (Table 5).

**Table 4:** Linear trends (mm/year, ℃/ year) of precipitation/temperature and perception of changes in climate. (RCC: Report of Climate Change; RI: Report Increased Precipitation/Temperature; RD: Report Decreased Precipitation/ Temperature).

	Jiuquan	Yongchang	Wshanling	Sunan	Minle	RCC (%)	RI (%)	RD (%)
Sum Pre	1.65	1.2	2.81	1.04	1.9	77	63	14
Win Pre	0.03	0.026	0.29	0.24	0.175	73	66	7
Sum Tem	0.04	0.6	0.04	0.03	0.045	83	75	8
Win Tem	0	0.01	0.005	0.014	0.022	69	54	15

**Table 5:** Most influential factors determining agro-pastoralists’ perception of climate change.

Variables	Coefficient	Robust Std. Err	*p*-value
Gender	0.325*	0.13	0.02
Age	0.075	0. 22	0.13
Experience	0.174*	0.043	0.03
Ethnicity	−0.653	0.47	0.21
Education	0.069*	0.031	0.02
household size	0.19	0.44	0.73
Cultivatedland size	0.023 **	0.14	0.01
Grassland size	0.23	0.59	0. 3
Income	0.29	0.74	0.56
Agricultural income	0.01*	0.00	0.03
Livestock	0.001*	0.00	0.02
Credit loan	1.578	0.53	0.32
Insurance	1.13	0.65	0.14
Association membership	0.542	0.34	0.00
Village cadres	0.309**	0. 16	0.00
Weather information	0.233**	0.12	0.00

**p < 0.01, *p < 0.05.

Significant values are in bold.

### Agro-pastoralists’ sources of climate change information

**Figure 6:**
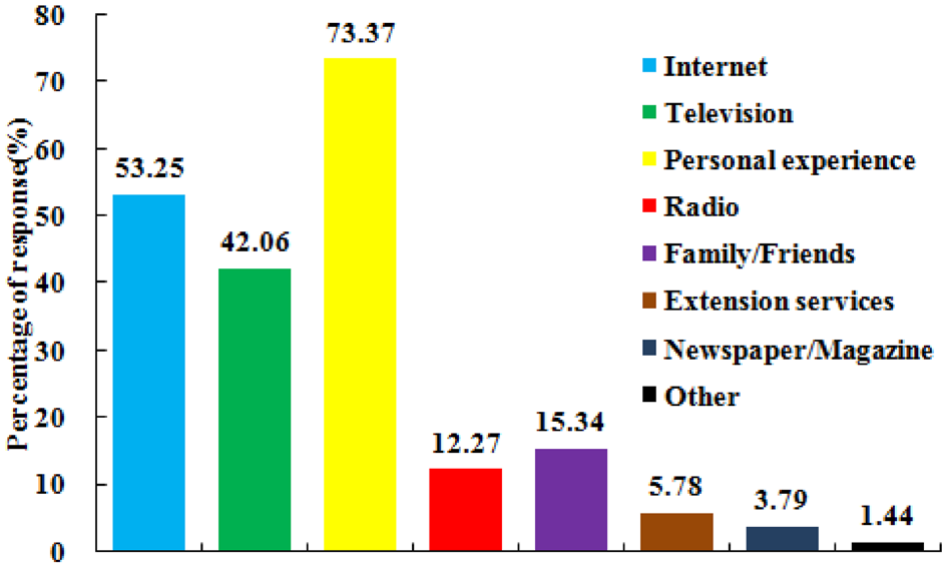
Agro-pastoralists’ sources of information, about climate change.

Availability and accessibility of information on climate change are assumed to be key determinants of the extent of agro-pastoralist awareness, understanding and knowledge of climate change impacts^54^. This research, therefore, explored the different ways that agro-pastoralists received information about climate change. The results are reported in Fig. 6. Figure 6 reveals that the respondents received information about climate change mainly from personal experience (73.37%), internet (53.25%), television (42.06%). Other sources such as radio (12.27%), family/friends (15.34%), association/extension (5.78%), newspaper/magazine (3.79%) and Other (1.44%) were far less important. Recently, communication devices (e.g., mobile phones, computers) that provide access to the internet play an important role for socioeconomic development in agro-pastoralist areas and may be relevant to spread information about climate change among agro-pastoralists.

### Determinants of agro-pastoralists’ perception

In order to further understand the relationship between Agro-pastoralist population characteristics and climate change perception, multiple regression (probit) analysis was used (Table 5).With the increase of experience, education, cultivatedland size, agricultural income, livestock, village cadres, access to weather information, agro-pastoralists’ awareness of climate change has increased significantly. The results also showed that women's perception of climate change was higher than men's.

The gender of respondents is a significant explanatory determinant (*p*-value is 0.02, coefficient is 0.325). In other words, women have more information and experience than men. This may be because in agricultural production, particularly in the planting and livestock sectors, women work significantly longer hours than men and are more sensitive to aware of the impacts from climate change on livelihoods^55^. The dependent variable significance of farming experience and their awareness of climate change (*p*-value 0.000, coefficient positive 0.39) indicates that experienced agro-pastoralists are more aware of climate change than inexperienced agro-pastoralists. These results are consistent with the findings of Eric et al.^25^.These results are consistent with the findings of Funatsu et al.^56^. The variables of respondents’ education level were highly significant (*P* value 0.01, coefficient 0.023). This means that agro-pastoralists are exposed to more climate events as their education level improves. This may be because well-educated agro-pastoralists are more sensitive to climate change because they are more scientific and technologically literate. These results are consistent with the findings of Maddison^57^ and Fahad et al.^58^.

The variables cultivatedland size, agricultural income and livestock have indicated a significant relationship with dependent variable at (*p*-value 0.01 and coefficient 0.023), (*p*-value 0.03 and coefficient 0.01) and (*p*-value 0.02 and coefficient 0.001), respectively. This reveals that with the increase of cultivatedland size, agricultural income and livestock, agro-pastoralists are more likely to be aware of climate change. These results are consistent with the findings of Oduniyi^59^, Mudombi et al.^60^ and Huong et al.^61^.

Village cadres and access to weather information variables have showed (coefficient 0.309, 0.233 and *p*-value 0.00, 0.00, respectively), which indicate that the agro-pastoralists who have the experience of serving as village cadres and have sufficient information or knowledge about climate change have higher awareness of climate change. Compared with ordinary agro-pastoralists, village cadres have a higher level of education and a richer social network, which can help collect and analyze information and improve understanding. In the interviewed areas, the main sources of information mentioned by agro-pastoralists are personal experience, internet and television. Our research findings are in line with Deressa et al.^20^ and Pondorfer^62^.

### Agro-pastoralists’ perceptions of climate change impacts

The research investigated how respondents perceived the impact of climate change on their livelihoods, especially on crop planting area, pasture quality, housing security, livestock loss, crop/livestock diseases, harvest time, seeding/calving time and agricultural income. Respondents were asked to explain to what extent variability in rainfall and temperature affected their livelihoods. The response frequency is reported in Table 6 (where 5 = High, 4 = Medium, 3 = low, 2 = No and 1 = Don't know).

**Table 6:** Agro-pastoralists’ perceptions of climate change impacts.

Variables	High	Medium	low	No	Don’t know
Crop area	15.8	29.27	42.5	10.09	2.34
Pasture quality	**38.4**	**21.82**	11.73	24.9	3.15
Housing security	**35.27**	**17.6**	12.36	30.09	4.68
Livestock loss	**27.9**	**32.3**	15.74	18.74	5.32
Crop/livestock diseases	**29.6**	**34.6**	13.47	19.52	2.81
Harvest time	12.1	8.4	43.75	29.48	6.27
Seeding/calving time	7.8	16.9	31.26	41.91	2.13
Agricultural income	**22.3**	**35.6**	17.66	20.73	3.71

Significant values are in bold.

Respondents have different views on the impact of climate change (Table 6). More than 50% of respondents said that climate change had a medium or above impact on pasture quality housing security livestock loss crop/livestock diseases agricultural income, and these variables were greatly affected by climate change. As for the planting area, the agro-pastoralists who are mainly planting are the most affected. This might be due to the high rainfall variability on agriculture production. Qin et al. (2004)^63^ estimated that by 2030, cereal production in China will decrease by 5–10% because of climate change. Liu et al. (2020)^64^ also reported that climate (i.e., rainfall and temperature) variability significantly impacted cereal production.

Among the respondents, nearly 15% disclosed that they have abandoned part of the land or are ready to abandon it; With regard to harvest time and harvesting / calving time, more than 70% said that the impact was small or not, but the survey showed that nearly 10 people said that the seeding time was ahead of schedule, and the calving time also changed, which may be related to the introduction of new varieties. Relevant research indicated that a significant portion of the negative impact of climate change on crops could be offset by growing new cultivars that had higher thermal time requirements or by implementing sowing date changes^65–69^.

### Factors affecting agro-pastoralists' adaptation to climate change

The probit model was used to evaluate the impact of selected variables on livelihood adaptability to the impact of climate change. A summary of the results is given in Table 7. The results in Table 7 show that the age, education, household size, grassland size, agricultural income, Association membership and village cadres significantly affect the adaptability of respondents.

**Table 7:** Most influential factors determinants agro-pastoralists’ adaptation to climate change.

Variables	Coefficient	Robust Std. Err	p-value
Gender	− 0.023	0.03	0.57
Age	0.047	0.37	0.11
Experience	0.053*	0.04	0.03
Ethnicity	− 0.13	0.09	0.82
Education	0.081**	0.04	0.00
household size	− 0.31*	0.16	0.04
Cultivatedland size	0.02	0.00	0.08
Grassland size	0.00**	0.00	0.00
Income	− 0.074	0.29	0.37
Agricultural income	0.017*	0.01	0.03
Livestock	0.037	0.40	0.12
Association membership	0.878*	0. 24	0.02
Village cadres	1.073**	0. 36	0.00
Weather information	0.915	0.31	0.09

Significant values are in bold.

Experience has a positive and significant impact on adaptation to climate change. Probit analysis showed that experience increased the adaptation similarity of respondents by 5.3%. Households headed with rich agricultural experience are more likely to adapt to the impact of climate change. Our findings are consistent with Maddison (2007)^57^, Ojo et al. (2021)^70^, Shahid et al. (2021)^71^, and Thoai et al. (2018)^72^, who revealed that farming experience and relevant adaptation measures have a significant impact on the adoption of adaptation measures.

Education is considered an important factor in adapting to climate change. Table 7 shows that education has a significant positive impact on respondents' adaptation to climate change impacts. An increase in formal education by one level was associated with a 8.1% increase in the likelihood of family adaptation. Our findings are consistent with Fosu-Mensah et al. (2012)^73^, Khanal et al. (2018)^74^, and Thoai et al. (2018)^72^, 2018, who revealed that education level is an important factor affecting the possibility of agro-pastoralists' adaptation to climate change.

The variable of household size shows that the ability of respondents to adapt to climate change increases with the increase of household size. Our findings are consistent with Khanal et al. (2019)^74^, Jha and Gupta (2021)^75^, and Shahid et al. (2021)^71^, who revealed that household size affects respondents' adaptive decision-making. However, our findings are in disagreement with Fahad et al. (2020)^58^, who reported an inverse relationship of household size with adaptability.

Table 7 shows a significant positive correlation between Grassland size, Agricultural income and adaptation. Respondents with a high Grassland size are more likely to adapt to climate change. The higher Agricultural income is, the more chance the family has to adapt to climate change. Our findings are consistent with Roco et al. (2014)^76^, and Arunrat et al. (2017)^77^, who revealed that household size affects respondents' adaptive decision-making.

Association membership positively and significantly affects adaptation to climate change impacts. Table 7 shows that Association membership has a significant positive impact on respondents' adaptation to climate change. When one Association is added, the family's adaptive capacity increases by 87.8 percent. Our findings are consistent with Roco et al. (2014)^76^, and Johnson and Brown (2017)^78^, who revealed that intensity of adaptation is greatly affected by farm organizations or associations.

The experience of village cadres has a significant positive effect on climate change adaptation. When one Association is added, the family's adaptive capacity increases by 87.8 percent.

The experience of village cadres has a significant positive effect on climate change adaptation. If there is village cadre experience, adaptability increases by more than one. It is easier for village cadres to take advantage of their positions to obtain information and take adaptive measures.

In order to reduce the negative impacts of climate change and maintain the livelihoods of rural households, it is necessary to improve the education level of agro-pastoralists, strengthen the capacity of community organization building and information dissemination services, and help agro-pastoralists better implement appropriate climate change management strategies.

### Adaptation strategies adopted by agro-pastoralists

**Figure 7:**
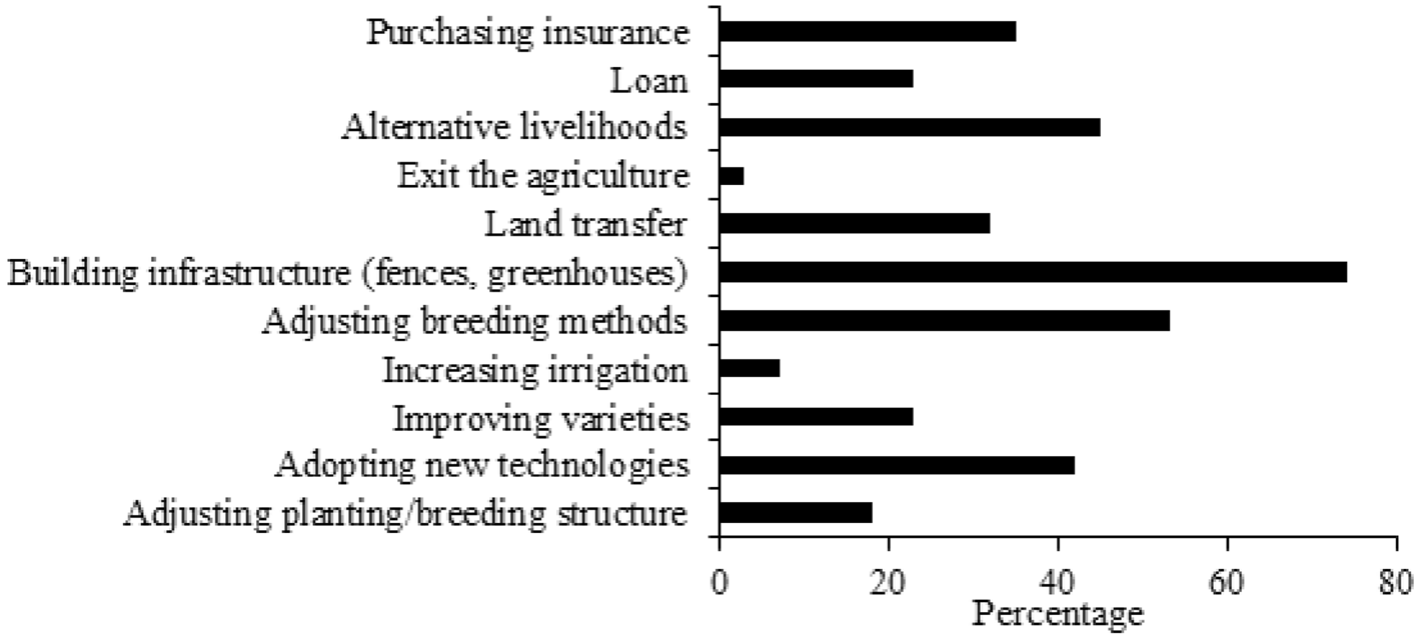
Agro-pastoralists’ adaptation strategies to climate change.

It was also anticipated that the survey would reveal whether agro-pastoralists had taken some strategies to cope with climate change. Agro-pastoralists were enquired as to whether they had adopted adaptation measures to respond to the impact of climate change. The adaptation strategies included adjusting planting/breeding structure, adopting new technologies, improving varieties, increasing irrigation, adjusting breeding methods, building infrastructure (fences, greenhouses), land transfer, exit the agriculture, out for work, loan, purchasing insurance (Fig. 7). It is clear from Fig. 7 that the most commonly used adaptation practices were building infrastructure (fences, greenhouses) and adjusting breeding methods, whereas increasing irrigation and exit the agriculture were the least adopted adaptation strategies. Moreover, adjusting breeding methods as well as alternative livelihoods were also introduced to respond to the impact of climate change. Among the surveyed agro-pastoralists, there were still those who did not adopt any strategy in response to climate change.

It should also be noted that agro-pastoralists who claimed to have observed climate change provide many reasons for their failure to take effective measures to cope with climate change, including lack of information, lack of funds, lack of labor, lack of land, and insufficient irrigation potential. The survey showed that there were two major obstacles: lack of funds and lack of information. These findings were consistent with Ojo and Baiyegunhi (2020)^79^, and Deressa et al. (2009)^20^. At present, the market price of agricultural products is very low compared to the cost of production, so agro-pastoralists are not willing to spend money and time in agricultural production, preferring to develop alternative livelihoods to increase their income.

## Conclusion

Agro-pastoralists' perception level is affected by several factors, such as socioeconomic factors and demographic factors. In this study, a random sampling method was used to sample 565 agro-pastoralists from four counties of Gansu province in northwest China. Our results show that the majority of respondents in the survey area (study area) are aware of climate change. The findings indicate that respondents who are severely affected by the adverse effects of climate change are more likely to be aware of climate change. The main factors that significantly affect respondents' understanding of climate change are gender, Farming experience, education level, cultivated land size, agricultural income, Livestock, village cadre experience, access to weather information. We further investigated agro-pastoralists' perception of climate change and their adaptation behaviors. Among the variables of study participants and adaptability, farming experience, education level, household size, grassland size, agricultural income, association membership, village cadre experience is closely related to the adaptability of agricultural and pastoral personnel. In addition, agro-pastoralists surveyed indicated that they had adopted aggressive adaptation strategies. The main reasons that hindered agro-pastoralists from taking effective measures to cope with climate change include lack of information, lack of funds, lack of labor, lack of land, and insufficient irrigation potential. Furthermore, our analysis of respondents' adaptive and perceived behavior provides a comprehensive understanding of the importance of climate in determining the effectiveness of agricultural programs.

Moreover, our findings could help government officials and agricultural extension workers expand their services and raise awareness of climate change in areas most vulnerable to climate hazards. On the government level, a top-down approach aimed at raising awareness of climate change may be appropriate if guided by common factors such as education or improved access to information and women's social status. Since the perception of climate change affects the adaptation choices of Agro-pastoralists^71^, we suggest that climate change education and public awareness should be strengthened, especially in rural communities with serious misunderstandings and limited farmers' ability to adapt to climate change.

While the study was limited to Gansu province in northwest China, the results of the study could be applied to other regions where adaptation to climate change remains ineffective. The study's recommendations help policy makers and researchers develop the necessary strategies and provide respondents with facilities to deal with climate risks. These efforts could focus on deep-seated adaptation measures, such as the use of high-yielding varieties and diversification of agro-pastoral operations. To strengthen such actions, policies and adequately supported approaches are needed to strengthen the capacity of agro-pastoralists to invest in adaptation strategies. It is suggested that agro-pastoralists' understanding of climate change and adaptation measures should be supported by mass media and information technology through agricultural and animal husbandry associations.
